# Multiplex Ultrasensitive Genotyping of Patients with Non-Small Cell Lung Cancer for Epidermal Growth Factor Receptor (EGFR) Mutations by Means of Picodroplet Digital PCR

**DOI:** 10.1016/j.ebiom.2017.06.003

**Published:** 2017-06-07

**Authors:** Masaru Watanabe, Tomoya Kawaguchi, Shun-ichi Isa, Masahiko Ando, Akihiro Tamiya, Akihito Kubo, Hideo Saka, Sadanori Takeo, Hirofumi Adachi, Tsutomu Tagawa, Osamu Kawashima, Motohiro Yamashita, Kazuhiko Kataoka, Yukito Ichinose, Yukiyasu Takeuchi, Katsuya Watanabe, Akihide Matsumura, Yasuhiro Koh

**Affiliations:** a Third Department of Internal Medicine, Wakayama Medical University, Wakayama, Japan; b Department of Respiratory Medicine and Medical Oncology, National Hospital Organization Nagoya Medical Center, Aichi, Japan; c Department of Respiratory Medicine, Graduate School of Medicine, Osaka City University, Osaka, Japan; d National Hospital Organization Kinki-Chuo Chest Medical Center, Osaka, Japan; e Clinical Research Center, Department of Thoracic Oncology, National Hospital Organization Kinki-Chuo Chest Medical Center, Osaka, Japan; f Center for Advanced Medicine and Clinical Research, Nagoya University Hospital, Aichi, Japan; g Department of Internal Medicine, National Hospital Organization Kinki-Chuo Chest Medical Center, Osaka, Japan; h Division of Respiratory Medicine and Allergology, Aichi Medical University School of Medicine, Aichi, Japan; i Department of Thoracic Surgery, Clinical Research Institute, National Hospital Organization Kyushu Medical Center, Fukuoka, Japan; j Department of Thoracic Surgery, National Hospital Organization Hokkaido Cancer Center, Hokkaido, Japan; k Department of Thoracic Surgery, National Hospital Organization Nagasaki Medical Center, Nagasaki, Japan; l Department of Thoracic Surgery, National Hospital Organization Shibukawa Medical Center, Gunma, Japan; m Department of Thoracic Surgery, National Hospital Organization Shikoku Cancer Center, Ehime, Japan; n Department of Thoracic Surgery, National Hospital Organization Iwakuni Clinical Center, Yamaguchi, Japan; o Clinical Research Institute, National Hospital Organization Kyushu Cancer Center, Fukuoka, Japan; p Department of General Thoracic Surgery, National Hospital Organization Toneyama National Hospital, Osaka, Japan; q Department of Thoracic Surgery, National Hospital Organization Yokohama Medical Center, Kanagawa, Japan; r Department of Surgery, National Hospital Organization Kinki-Chuo Chest Medical Center, Osaka, Japan

**Keywords:** NSCLC, non–small cell lung cancer, TKI, tyrosine kinase inhibitor, PFS, progression-free survival, SARMS, Scorpion Amplification Refractory Mutation System, dPCR, digital polymerase chain reaction, ddPCR, picodroplet dPCR, mCRC, metastatic colorectal cancer, JME, Japan Molecular Epidemiology for Lung Cancer, FFPE, formalin-fixed paraffin-embedded, TET, tetrachlorofluorescein, FAM, 6-carboxyfluorescein, LOB, limit of blank, *EGFR* mutation, Droplet digital PCR, Non–small cell lung cancer

## Abstract

Epidermal growth factor receptor (EGFR) mutations have been used as the strongest predictor of effectiveness of treatment with EGFR tyrosine kinase inhibitors (TKIs). Three most common *EGFR* mutations (L858R, exon 19 deletion, and T790M) are known to be major selection markers for EGFR-TKIs therapy. Here, we developed a multiplex picodroplet digital PCR (ddPCR) assay to detect 3 common *EGFR* mutations in 1 reaction. Serial-dilution experiments with genomic DNA harboring EGFR mutations revealed linear performance, with analytical sensitivity ~ 0.01% for each mutation. All 33 EGFR-activating mutations detected in formalin-fixed paraffin-embedded (FFPE) tissue samples by the conventional method were also detected by this multiplex assay. Owing to the higher sensitivity, an additional mutation (T790M; including an ultra-low-level mutation, < 0.1%) was detected in the same reaction. Regression analysis of the duplex assay and multiplex assay showed a correlation coefficient (R^2^) of 0.9986 for L858R, 0.9844 for an exon 19 deletion, and 0.9959 for T790M. Using ddPCR, we designed a multiplex ultrasensitive genotyping platform for 3 common *EGFR* mutations. Results of this proof-of-principle study on clinical samples indicate clinical utility of multiplex ddPCR for screening for multiple EGFR mutations concurrently with an ultra-rare pretreatment mutation (T790M).

## Highlights

• An ultrasensitive multiplex picodroplet digital PCR (ddPCR) assay to detect 3 common EGFR mutations in 1 reaction was developed.
• This multiplex mutation detection of common mutations of EGFR is a feasible alternative.
• A rare pretreatment EGFR mutation (T790M) with a mutant allele frequency below 0.1% was detected via this multiplex assay.

Three most common EGFR mutations (L858R, exon 19 deletion, and T790M) are known to be major selection markers for EGFR-TKI therapy. In this article, we developed a multiplex picodroplet digital PCR (ddPCR) assay to detect 3 common EGFR mutations in 1 reaction with 0.01% sensitivity. All 33 EGFR-activating mutations detected in FFPE samples by the conventional method were also detected by this multiplex assay. Owing to the higher sensitivity, an additional mutation was detected in the same reaction. Results of this proof-of-principle study on clinical samples indicate clinical utility of multiplex ddPCR for screening for multiple EGFR mutations.

## 1 Introduction

Targeted molecular therapy has improved the treatment of non–small cell lung cancer (NSCLC). Superiority of epidermal growth factor receptor (EGFR) tyrosine kinase inhibitors (TKIs) to platinum-based chemotherapy in terms of progression-free survival (PFS) in EGFR-mutated lung cancers has been reported in several phase III trials as a first-line treatment (Zhou et al., 2011, Rosell et al., 2012, Mok et al., 2009, Mitsudomi et al., 2010, Maemondo et al., 2010). EGFR-TKIs (gefitinib, erlotinib, or afatinib) have been demonstrated to be effective for NSCLC patients with EGFR-activating mutations such as exon19 deletion or exon 21 L858R mutations (Lynch et al., 2004, Paez et al., 2004). Evidence shows, however, that most responders eventually develop acquired resistance to EGFR-TKIs (Kobayashi et al., 2005, Yu et al., 2013, Ohashi et al., 2013). Among these patients, a secondary missense T790M mutation is observed in nearly half of all cases resistant to EGFR-TKIs (Ohashi et al., 2013).

This T790M mutation was also detected in tumors as a minor cellular clone before exposure to EGFR-TKIs and was found concurrently with other EGFR-activating mutations (Inukai et al., 2006). This “pretreatment T790M mutation” is present in 1–8% of cases according to conventional DNA sequencing like Sanger sequencing (Wu et al., 2011, Sequist et al., 2008, Li et al., 2014, Fujita et al., 2012) and in 2–79% of cases according to more sensitive detection methods like Scorpion Amplification Refractory Mutation System (SARMS) technology with an EGFR-activating mutation (Su et al., 2012, Rosell et al., 2011, Maheswaran et al., 2008, Costa et al., 2014, Yu et al., 2014). Patients with pretreatment T790M mutation detected by less sensitive methods show a lower response rate and shorter PFS (Inukai et al., 2006, Wu et al., 2011, Sequist et al., 2008). Recent studies revealed that patients with a pretreatment T790M mutation detected by a highly sensitive method also have shorter PFS (Su et al., 2012, Rosell et al., 2011, Maheswaran et al., 2008, Costa et al., 2014, Ding et al., 2014), suggesting that a low-level pretreatment T790M mutation can be used for optimizing treatment with EGFR-TKIs. Therefore, the ability of molecular analytical technologies to detect EGFR mutants at the subclone level before EGFR-TKI treatment is critically important for enabling more personalized therapies in NSCLC.

Picodroplet digital PCR (ddPCR) recently emerged as a highly sensitive method for detection of gene mutations and is based on compartmentalization of DNA into picoliter-size droplets (Taly et al., 2012). Our previous report showed detection of 0.001% prevalence of the *EGFR* T790M mutation among tumor cells (Watanabe et al., 2015). Several examples of ddPCR application to highly sensitive detection of mutations were published recently (Pekin et al., 2011, Oxnard et al., 2014, Ono et al., 2014, Iwama et al., 2015, Sacher et al., 2016). Multiplexing of mutation detection in a single assay is desirable for genotype testing in the clinic; promising results have also been demonstrated using ddPCR (Zhong et al., 2011, Didelot et al., 2013, Taly et al., 2013, Laurent-Puig et al., 2015, Zonta et al., 2016). The multiplex procedure has been adapted to quantitative detection of 7 common mutations of *KRAS* (in codons 12 and 13) in plasma samples and primary tumor samples from patients with metastatic colorectal cancer (mCRC) (Taly et al., 2013, Laurent-Puig et al., 2015). Zonta et al., developed several multiplex panels for EGFR (several three- and four-plex) in reference standard DNA samples. Here, we report the advantage of our 6-plex ddPCR assay that detects 3 clinically relevant mutations of EGFR (L858R, exon 19 deletion, and T790M mutations) and corresponding wild-type allele at an ultra-low level by using DNA samples of surgically resected primary tumors from 45 NSCLC patients.

## 2 Materials and Methods

### 2.1 Study Design and Patients

We used this test system to assess multiplex detection of 3 *EGFR* mutations in 45 samples of surgically resected primary tumors from NSCLC patients enrolled in the Japan Molecular Epidemiology for Lung Cancer Cases (JME) study (Kawaguchi et al., 2016). That study (UMIN000008177) is a prospective, multicenter molecular epidemiological analysis designed to address associations between driver mutations and smoking and other environmental factors. Eligible subjects are patients with newly diagnosed NSCLC of stage I to IIIB who have received surgical treatment. Full details of the study design were published elsewhere (Kawaguchi et al., 2016).

The present study was approved by the Institutional Review Board of the National Hospital Organization of Japan. All patients provided written informed consent. From July 2012 to December 2013, 958 patients were recruited from 43 institutions, and 901 samples were successfully analyzed.

Genomic DNA extraction from formalin-fixed, paraffin-embedded (FFPE) specimens of surgically resected tissue was performed in an independent clinical laboratory (SRL, Tokyo, Japan). Genomic DNA concentration was measured using the PicoGreen dsDNA quantitation assay (Life Technologies, Carlsbad, CA) as per the manufacturer's recommendation. Fluorescent intensity from double strand DNA was measured by GloMax-Multi Microplate Multimode Reader (Progega, Madison, WI). Somatic mutations in *EGFR* and *KRAS* were validated by sensitive PCR methods in an independent clinical laboratory (SRL).

### 2.2 DNA Controls

Positive and negative control plasmids for the EGFR assay were prepared by cloning DNA fragments containing wild-type or the EGFR mutations were using a TOPO TA Cloning Kit (Life Technologies). The appropriate concentration of plasmid DNA was determined empirically to yield a mixture in which the number of copies of mutant DNA was ca. 0.01–1.000% of the number of wild-type EGFR fragments.

Tumor cell lines H1975, PC-9/ZD, and A549 are a gift from Dr. Fumiaki Koizumi (Tokyo Metropolitan Cancer and Infectious Diseases Center Komagome Hospital, Tokyo, Japan). Genomic DNA was extracted using a QIAamp DNA Mini Kit (Qiagen, Hilden, Germany). Wild-type human genomic DNA was purchased from Clontech (Mountain View, CA). Genomic-DNA samples were digested with CviQ1 (New England Biolabs, Ipswich, MA), and DNA concentration was determined using a Qubit® fluorometer (Life Technologies). Digested-DNA controls were used to quantitatively assess each EGFR mutant sequence and in the multiplex assay panels. In total, 400 ng digested DNA was used for control experiments to determine the limit of blank (LOB).

Genomic DNA of each mutation-specific cell line was serially diluted with wild-type human genomic DNA to attain mutation prevalence between 0.01% and 1%. Evaluation of the linearity and lower limit of mutation detection of each probe was also performed for multiplex ddPCR assays.

### 2.3 Probes and Primers for Digital PCR

Primers and probes were acquired from MBL-IDT K.K (Nagoya, Japan). Fluorescent probes targeting wild-type and mutant sequences were respectively conjugated to tetrachlorofluorescein (TET; λ_ex_ 522 nm, λ_em_ 539 nm) or 6-carboxyfluorescein (FAM; λ_ex_ 494 nm, λ_em_ 522 nm) fluorophores with the ZEN/IABkFQ double quencher. Sequences of primers and probes for detection of *EGFR* mutations are given in Supplemental Table 1.

### 2.4 *EGFR* Mutation Detection

This duplex assay is based on parallel amplification of wild-type and specific mutant sequences. In a pre-PCR setup, 20.0 μL (mm^3^) TaqMan Genotyping Master Mix (Life Technologies) was mixed with the assay solution containing 2.0 μL of 10 μM (i.e., 10^− 2^ × mol/m^3^) forward and reverse primers, 2.0 μL of 4 μM FAM and TET labeled-probes, 4.0 μL Droplet Stabilizer (RainDance Technologies, Billerica, MA), 4.0 μL sterile DNase- and RNase-free water (Life Technologies), and 4 μL genomic DNA from patients (57.7–311.6 ng), with a final reaction volume of 40 μL.

The multiplex assay was developed to identify 3 common *EGFR* mutations and each corresponding wild-type sequence. The final reaction volume was 40 μL, with 4 μL genomic-DNA samples from patients. Final concentrations of primers and probes are shown in Supplemental Table 1.

### 2.5 Emulsification and Thermal Cycling of the Emulsion

A collection of uniformly sized aqueous droplets was produced by hydrodynamic flow focusing with a droplet-generating microfluidic chip (Souse chip, RainDance). The resulting emulsion was collected into a PCR tube strip comprising eight 0.2-mL conical-bottom PCR tubes (Axygen, Tewksbury, MA). The PCR tube strip, containing 75 μL droplets and carrier oil per tube, was tightly capped with an 8-Strip Dome Cap (Axygen), and then placed in a thermal cycler with a hot lid (Proflex PCR System, Life Technologies). The emulsion was subjected to thermal cycling under conditions described in Supplemental Table 2.

After that, the emulsion was transferred into a second microfluidic chip (Sense chip, RainDance), and endpoint fluorescence signals were measured.

### 2.6 Data Analysis

The droplet event data were analyzed in the RainDrop Analyst software (RainDance) following manufacturer's instructions. Briefly, sample data were loaded with a drop size gating template (RainDance). Data from the positive control sample were used to create the compensation matrix in the RainDrop Analyst software. The compensation matrix was applied to data from each sample to eliminate crosstalk fluorescence signals from the TET and FAM fluorophores. Sizes and locations of wild-type- and mutant-specific gates were established by manual selection of the area containing wild-type- or mutant-specific clusters in the positive control.

For each unknown sample, PCR-positive droplet events were counted within each gate. The number of events within each gate was converted to the number of events per assay using the total number of intact drops.

### 2.7 Statistical Analysis

All statistical analyses were performed by means of the Prism software (GraphPad Software Inc., La Jolla, CA).

## 3 Results

### 3.1 Use of Multiplex ddPCR to Detect 3 Common *EGFR* Mutations

**Fig. 1:**
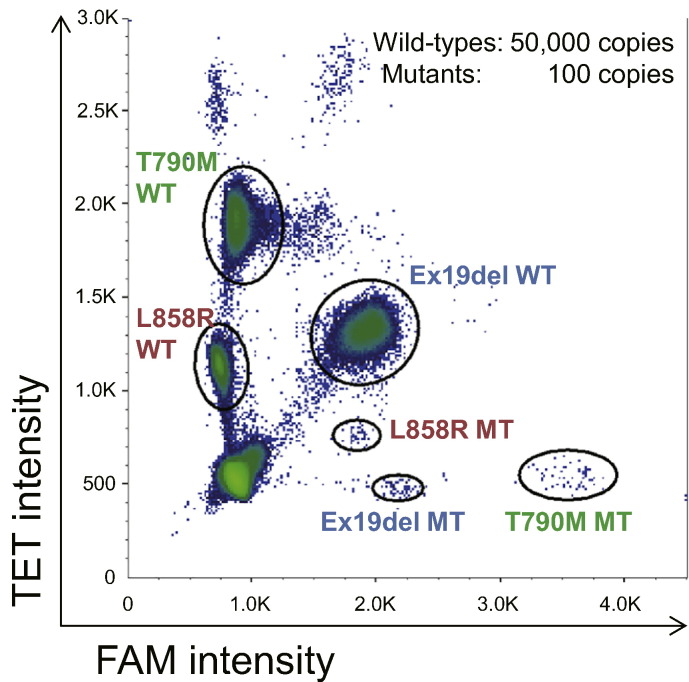
Multiplex assay for analysis of *EGFR* mutations. Two-dimensional histogram of the 6-hexaplex assay is shown. Plasmids containing each target sequence were encapsulated in droplets and subjected to the ddPCR assay. FAM, 6-carboxyfluorescein; TET, tetrachlorofluorescein.

A multiplex assay requires detection of multiple mutations concurrently in a single assay. Such a test minimizes the assay cost and amount of biological samples and allows researchers to measure mutation frequency among DNA molecules accurately for each mutation (Zhong et al., 2011, Didelot et al., 2013, Taly et al., 2013, Laurent-Puig et al., 2015, Zonta et al., 2016). The fluorescence intensity of end-point PCR may depend on the nature and concentration of the fluorescent probe, which enables researchers to distinguish and quantify droplets containing each specific target. Each target population appears as a distinct cluster of droplets in a 2-dimensional histogram. Assays for each of *EGFR* mutations under study were constructed by mixing mutation-specific FAM and/or TET fluorescent probes with the corresponding wild-type-specific TET probes and 3 pairs of PCR primers (Supplemental Table 1). The concentrations of the probes were optimized to distinguish among empty droplets, droplets containing wild-type EGFR DNA, and droplets containing DNA with a specific *EGFR* mutation (Supplemental Table 1). As shown in Fig. 1, the hexaplex assay panel revealed the presence of *EGFR* L858R, exon 19 deletion, or T790M mutations and each corresponding wild-type. To improve probe discrimination toward *EGFR* L858R, a TET-labeled mutation-specific probe was added to the reaction (Supplemental Table 1).

### 3.2 Assessment of the Multiplex ddPCR Assay

To assess performance of our multiplex ddPCR assay, a plasmid containing a mutant sequence was added to the solution of the plasmid containing a wild-type sequence, and then the multiplex ddPCR assay was performed. Results (2-dimensional histogram) of the multiplex ddPCR assay are summarized in Supplemental Fig. 1A. Regression analysis of the observed mutant allele proportion (%) versus the expected mutant allele proportion (%) yielded correlation coefficients (R^2^) of 0.9997, 0.9999, and 0.9999 for L858R, an exon 19 deletion, and T790M, respectively (Supplemental Fig. 1B).

**Fig. 2:**
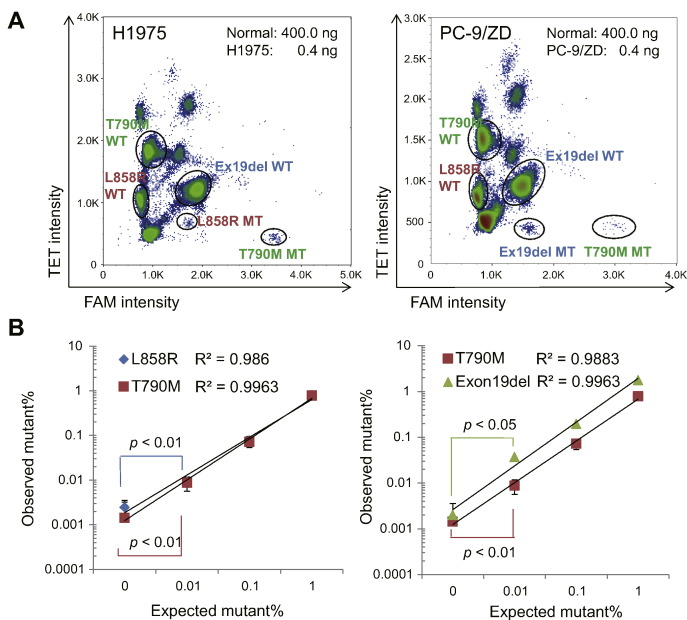
Serial dilutions of positive controls were analyzed for each *EGFR* mutation under study. Testing 0.04 and 4 ng of genomic DNA from H1975 cells harboring *EGFR* mutations L858R and T790M (A) or PC-9/ZD cells harboring EGFR exon 19 deletion and substitution T790M (B); n = 5.

We next tested whether the multiplex assay can identify the specific mutation in genomic DNA from tumor cells harboring *EGFR* mutations. The H1975 and PC-9/ZD human lung tumor cell lines carry *EGFR* mutations L858R + T790M and exon 19 deletion + T790M, respectively. Genomic DNA samples from those cell lines were serially diluted with human normal genomic DNA across 3 logarithms of concentrations of the mutants. Each dilution was analyzed with the multiplex panel, and results of the 2-dimensional histogram from the multiplex ddPCR assay are summarized in Fig. 2a. The measured allele frequency matched the expected allele frequency over the range of 1% to 0.01% for all three mutations (Fig. 2b), suggesting our multiplex ddPCR assay detects a mutation prevalence as low as 0.01%. Several events were counted in controls with no DNA present, suggesting these events are due to the counting of false-positive droplets and define the limit of detection.

The limit of blank (LOB) is the primary characteristic of an assay that determines the lower limit of detection, and the LOB was defined by the frequency of test-positive droplets in wild-type samples as well as in human normal genomic DNA. The number of false positive droplet events was measured for 8 negative control experiments by means of 200,000 copies of wild-type plasmid DNA controls, 400 ng of human normal genomic DNA, and 400 ng of genomic DNA from the EGFR wild-type A549 cell line (Supplemental Fig. 2). The rate of false positive droplet events did not depend on the total amount of DNA (data not shown, Taly et al., 2013, Kawaguchi et al., 2016). Therefore, the LOB was determined by evaluating the 95% one-tailed upper limit of the model distribution, as done in previous reports (Taly et al., 2013, Kawaguchi et al., 2016). The number of false positive events of mutant droplets detected per assay was 9 for L858R, 10 for an exon 19 deletion, and 7 for T790M in wild-type plasmid DNA; 8 for L858R, 6 for an exon 19 deletion, and 6 for T790M in human normal genomic DNA; and 9 for L858R, 4 for exon 19 deletion, and 2 for T790M in A549 genomic DNA (Supplemental Fig. 2D).

### 3.3 Multiplex Analysis of DNA from FFPE Samples of Surgically Resected Primary Lung Tumors

For each patient sample, the expected mutation status was determined in the primary tumor DNA via conventional Cycleave assays or the SARMS assay for EGFR or KRAS mutations, respectively. The mutation status distribution among the 45 tumor samples is presented in Table 1, Table 2, Table 3.

**Table 1:** Duplex and multiplex analyses of FFPE samples from patients with a tumor carrying the *EGFR* L858R mutation.

Sample#	Amount of input DNA (ng)	Tumor mutation (Cycleave method)	Duplex analysis	Multiplex analysis
			Mutation (%)	Mutation (%)
1	141.0	L858R, T790M	L858R (37.080), T790M (9.266)	L858R (35.221), T790M (9.194)
2	156.8	L858R, T790M	L858R (7.370), T790M (7.857)	L858R (7.090), T790M (7.742)
3	151.3	L858R, T790M	L858R (36.612), T790M (32.375)	L858R (33.305), T790M (36.615)
4	216.5	L858R, T790M	L858R (26.876), T790M (23.592)	L858R (24.583), T790M (23.761)
5	184.1	L858R	L858R (18.336), T790M (0.136)	L858R (16.841), T790M (0.171)
6	155.4	L858R	L858R (10.090), T790M (0.864)	L858R (8.465), T790M (0.818)
7	77.8	L858R	L858R (32.145), T790M (0.024)	L858R (29.869), T790M (0.030)
8	60.9	L858R	L858R (14.814), T790M (0.035)	L858R (13.335), T790M (0.027)
9	76.4	L858R	L858R (5.456)	L858R (5.011)
10	57.7	L858R	L858R (5.898)	L858R (6.056)
11	69.1	L858R	L858R (6.408)	L858R (6.387)
12	130.4	L858R	L858R (7.718)	L858R (7.171)
13	109.1	L858R	L858R (27.927)	L858R (27.717)
14	79.7	L858R	L858R (4.041)	L858R (3.876)
15	168.1	L858R	L858R (30.548)	L858R (29.174)
16	151.1	L858R	L858R (4.098)	L858R (4.351)

**Table 2:** Duplex and multiplex analyses of FFPE samples from patients with a tumor harboring an *EGFR* Exon 19 deletion.

Sample#	Amount of input DNA (ng)	Tumor mutation (Real-time PCR)	Duplex analysis	Multiplex analysis
			Mutation (%)	Mutation (%)
1	72.8	Ex19del, T790M	Ex19del (30.334), T790M (5.284)	Ex19del (29.686), T790M (5.382)
2	214.3	Ex19del	Ex19del (19.068), T790M (1.375)	Ex19del (17.930), T790M (1.440)
3	135.7	Ex19del	Ex19del (19.746), T790M (0.307)	Ex19del (22.187), T790M (0.302)
4	216.0	Ex19del	Ex19del (28.217), T790M (0.135)	Ex19del (31.216), T790M (0.117)
5	153.6	Ex19del	Ex19del (3.674), T790M (0.073)	Ex19del (4.131), T790M (0.091)
6	231.8	Ex19del	Ex19del (37.183), T790M (0.024)	Ex19del (37.896), T790M (0.026)
7	268.0	Ex19del	Ex19del (40.174), T790M (0.041)	Ex19del (38.940), T790M (0.030)
8	311.6	Ex19del	Ex19del (11.818), T790M (0.046)	Ex19del (12.522), T790M (0.054)
9	141.7	Ex19del	Ex19del (37.136), T790M (0.041)	Ex19del (38.142), T790M (0.040)
10	187.7	Ex19del	Ex19del (25.069)	Ex19del (22.298)
11	153.2	Ex19del	Ex19del (37.218)	Ex19del (35.742)
12	123.1	Ex19del	Ex19del (32.418)	Ex19del (32.788)
13	113.0	Ex19del	Ex19del (38.710)	Ex19del (37.505)
14	169.2	Ex19del	Ex19del (16.112)	Ex19del (15.451)
15	123.4	Ex19del	Ex19del (44.617)	Ex19del (43.660)
16	100.2	Ex19del	Ex19del (16.992)	Ex19del (15.538)
17	78.5	Ex19del	Ex19del (28.056)	Ex19del (28.622)

**Table 3:** Duplex and multiplex analyses of FFPE samples from patients with a *KRAS*-mutated tumor.

Sample#	Amount of input DNA (ng)	Tumor mutation (Real-time PCR)	Duplex analysis	Multiplex analysis
			Mutation (%)	Mutation (%)
1	215.2	KRAS G12A	No EGFR mutation detected	No EGFR mutation detected
2	116.8	KRAS G12V	No EGFR mutation detected	No EGFR mutation detected
3	165.5	KRAS G12D	No EGFR mutation detected	No EGFR mutation detected
4	194.9	KRAS G12C	No EGFR mutation detected	No EGFR mutation detected
5	134.8	KRAS G12D	No EGFR mutation detected	No EGFR mutation detected
6	125.2	KRAS G12C	No EGFR mutation detected	No EGFR mutation detected
7	236.4	KRAS G12V	No EGFR mutation detected	T790M (0.025)
8	141.2	KRAS G21A	No EGFR mutation detected	No EGFR mutation detected
9	226.6	KRAS G12A	No EGFR mutation detected	No EGFR mutation detected
10	163.2	KRAS G12D	No EGFR mutation detected	No EGFR mutation detected
11	94.8	KRAS G12V	No EGFR mutation detected	No EGFR mutation detected
12	161.3	KRAS G13D	No EGFR mutation detected	No EGFR mutation detected

According to LOBs determined using control DNA (Supplemental Fig. 2D), we first used 10 events/assay as our threshold for a positive result. Nonetheless, the rate of false positive droplet events in assays for L858R and exon 19 deletion in FFPE samples showed a moderate dependence on the total amount of DNA (Supplemental Fig. 3). Therefore, the LOB had to be expressed as a definitive proportion (%) of the mutant allele in those assays, and the determined threshold for assays of L858R and exon 19 deletion was 0.07% and 0.4%, respectively (Supplemental Fig. 4). In contrast, the rate of false positive droplet events in the T790M assay did not depend on the total amount of DNA due to the position of the end point signal (Supplemental Fig. 3). Thus, we defined 11 events/assay as a threshold for a positive result on the T790M mutation (Supplemental Fig. 4).

Of the 16 FFPE samples in which an *EGFR* L858R mutation was identified in the tumor, 16 were positive for the same mutation as that assessed in the multiplex ddPCR assay (Table 1). Four samples were also positive for an additional mutation (T790M) in the tumors; those 4 samples were also T790M-positive according to the multiplex ddPCR assay. Furthermore, 4 additional T790M mutations were found by means of the multiplex ddPCR assay and were confirmed by the duplex assay (described in the section below) to be present at an ultralow frequency, suggesting that the multiplex assay detected a rare mutation that was detected by the duplex assay but not the conventional assay. Of 17 FFPE samples in which an *EGFR* exon 19 deletion was identified in the tumor, 17 were positive for the same mutation as that detected by the multiplex ddPCR assay (Table 2). Nine additional mutations (T790M) were also detected with the multiplex ddPCR assay. Twelve FFPE samples in which a *KRAS* mutation without an *EGFR* mutation was detected by the duplex assay were expected to test negative (Table 3). Eleven were also test-negative according to the multiplex assay for a T790M mutation, with 1 sample that tested positive in the multiplex assay (Sample ID #0314, 13.5 copies/assay).

### 3.4 Duplex Assay vs. Multiplex Assay

**Fig. 3:**
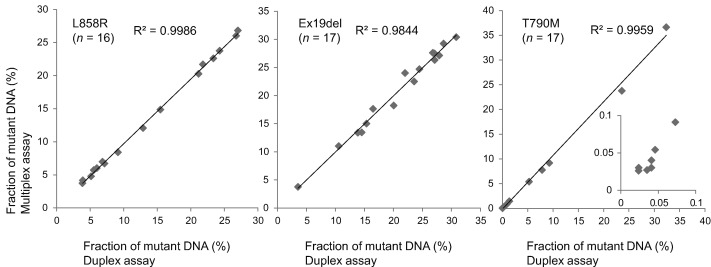
Comparison of results obtained by multiplex and duplex analyses. Compilation of the analysis of FFPE samples from patients with primary tumors mutated for EGFR L858R, Exon19 deletion (Ex19del) with/without T790M mutation.

To confirm results obtained with the multiplex ddPCR, we conducted additional analyses of the corresponding FFPE samples with a duplex ddPCR assay in which only 2 molecular targets (i.e., wild-type and a corresponding mutant allele) are detected in each assay for three *EGFR* mutations. Results of the duplex ddPCR analysis for these samples are summarized in Table 1, Table 2, Table 3, and 3 representatives are shown in Supplemental Fig. 5. Regression analysis yielded a correlation coefficient (R^2^) of each mutant allele (0.9986 for L858R, 0.9844 for an exon 19 deletion, and 0.9959 for T790M) (Fig. 3) as well as each wild-type allele (Supplemental Fig. 6), indicating that results of the multiplex EGFR assay were completely concordant with those of the duplex ddPCR assay.

## 4 Discussion

Here, we report a ddPCR-based ultrasensitive multiplex assay for the 3 common mutations of *EGFR* (L858R, exon 19 deletion, and T790M) in FFPE samples from NSCLC patients; this assay allows the detection of mutations in different exons with multiple primer sets via digital PCR on genomic DNA samples from tumor tissues. Our results suggest that multiplex mutation detection of common mutations of EGFR is a feasible alternative to a duplex assay for detecting EGFR mutations simultaneously. With our multiplex assay, we also detected a rare pretreatment EGFR mutation (T790M) with a mutant allele frequency below 0.1%. This mutation was not detected by commercially available methods, and our data were confirmed by a duplex assay. In this work, the high sensitivity and accuracy of multiplex picodroplet dPCR enabled detection and quantification of 2 EGFR-activating mutant alleles and a rare EGFR-TKI resistance allele in NSCLC tumor samples.

The mutational status of the patients was determined in the samples of primary tumors before the initiation of EGFR-TKI therapy. Somatic activating mutations of *EGFR* are associated with dramatic tumor-related therapeutic responses and favorable clinical outcomes for EGFR-TKIs in patients with NSCLC (Mitsudomi et al., 2010, Maemondo et al., 2010). The pretreatment *EGFR* T790M mutation is also important for efficacy of EGFR-TKIs. In several studies (Inukai et al., 2006, Wu et al., 2011, Sequist et al., 2008), a higher rate of the pretreatment T790M mutation according to low-sensitivity methods (such as direct sequencing) resulted in a lower response rate or worse PFS. It is noteworthy that the rare pretreatment T790M mutation detected by highly sensitive methods such as mutant enriched-PCR results in shorter PFS (Costa et al., 2014). Some third-generation EGFR-TKIs that target the *EGFR* T790M mutation have been evaluated in several clinical trials (Sequist et al., 2015, Jänne et al., 2015, Mok et al., 2016). These new drugs when used as a first-line treatment are expected to eradicate tumors harboring EGFR-activating mutations and pretreatment T790M mutations. For these purposes, our ultrasensitive multiplex ddPCR assay may be useful during screening for multiple *EGFR* mutations in 1 reaction to precisely diagnose the disease prior to EGFR-TKI treatment.

Sensitivity and multiplicity of mutation detection contradicted each other. The conventional quantitative-PCR-based dual-probe assays have previously been validated for clinical uses and showed the maximal sensitivity of ~ 0.1% (Yatabe et al., 2006, Milbury et al., 2009). These highly sensitive assays detect only 2 molecular targets (i.e., a single mutant and a corresponding wild type) in single reaction. Although next-generation-sequencing–based assays detect > 8000 single nucleotide variations in approximately 50 genes, maximal sensitivity (~ 2%) is much worse than that of quantitative-PCR-based assays (Kawaguchi et al., 2016). In the present study, we converted the duplex digital PCR assays to a multiplex format, which allowed for detection and quantification of multiple common *EGFR* mutations in 1 sample with ultrahigh sensitivity (~ 0.01%).

Detecting the 3 most common *EGFR* mutations is challenging for an amplification-based assay simultaneously in single reaction, because the 3 mutations are located in different exons, meaning that 3 primer sets are required. Previously, a multiplex assay for detecting adjacent codons with a single primer set was successful (Taly et al., 2013). Zonta et al. developed four-plex assay targeting the three common EGFR mutations with only one wild-type sequence. Our six-plex assay detects the three common EGFR mutations with all of corresponding wild-type sequences. This makes great difference in terms of accurate calculation of variant frequency of the mutant allele of interest. In fact, the results of our multiplex assay are identical to those of duplex assay in terms of not only variant call but also variant frequency (Table 1). In addition, Zonta et al. validated their four-plex assay using reference standard DNA samples in 20 to 60 ng range. In contrast, our six-plex assay was validated by using a large amount of input DNA (57.7–311.6 ng) extracted from clinical specimens.

There remains a technical problem with a large amount of input DNA and/or poor quality DNA. The number of false positive events for some assays is increased by poor discrimination of the end point signal from other clusters within a 2-dimensional histogram in a multiplex assay. The insufficient separation of clusters leads to lower sensitivity due to false positives. In fact, the fluorescence cluster associated with the assays for L858R has a limited space between the cluster associated with the assays for an exon 19 deletion and the corresponding wild-type droplets. The amount of false positive droplets was increasing with total amount of input DNA as noise near the clusters. Poor sample quality also yielded false positive droplet events. In the assay design for detecting an EGFR exon 19 deletion, when a wild-type DNA molecule is amplified, signals from the wild-type-specific probe and the reference probe can both be detected. If a mutant molecule with a deletion is amplified, only the signal from the reference probe can be detected (Yung et al., 2009). Thus, poor sample quality can produce artificial mutation calls due to interference with amplification of a wild-type-specific region. Consequently, the LOB for exon 19 deletion is relatively high. In contrast, the assay for detection of EGFR T790M provides better separation of clusters and more definitive identification of true positive droplet events. One sample, however, was miscalled by the multiplex assay in comparison with the duplex assay. A sample bordering the threshold in the multiplex assay should be confirmed by the duplex assay (10 to 20 copies/assay).

Our 6-plex assay has a great advantage in dealing with lower amount of DNA, such as plasma DNA and DNA from small biopsies. The FDA has approved the plasma DNA EGFR testing as a companion diagnostic for EGFR-TKIs and analytical sensitivity of the assay is 5% at lowest according to manufacturer's instructions. The sensitivity of our 6-plex assay is high enough to detect mutations in plasma samples, and false positive rate should be much lower than FFPR samples due to much lower amount of input DNA. Further evaluation to establish EGFR mutation detection using liquid samples or small biopsies is warranted. In conclusion, we developed an ultrasensitive multiplex assay for detecting 3 common *EGFR* mutations. Using this assay, we can suggest a clinically relevant threshold, which may help to decide whether to treat NSCLC patients with an EGFR-TKI. Our data show the potential of multiplex ddPCR as a high-resolution diagnostic tool for stratification of patients with the aim of more personalized treatment. Continuing improvement of cluster separation may help to reduce the number of false positive events and to increase the sensitivity. Based upon these initial results, further validation involving prospectively collected tumor and plasma samples should be conducted in the near future.

## Role of Funding Sources

This work was supported by a Grant-in-Aid for Japanese National Hospital Organization Multi-Center Clinical Research for Evidence-Based Medicine (H23-EBM-01), Japan and the Japanese Society for the Promotion of Science (JSPS) KAKENHI Grant Numbers 15K09226.

## Authors' Disclosures or Potential Conflicts of Interest

YK: Honoraria: RainDance Technologies.

## Author Contributions

MW and YK conceived and designed the experiments; MW developed methodology and performed the experiments; MW and YK analyzed the data and wrote the manuscript. TK, SI, MA, AT, AK, HS, ST, HA, TT, OK, MY, KK, YI, YT, KS, AM, and YK provided administrative, technical, or material support. All authors reviewed the results and approved the final version of the manuscript.

## Appendix A Supplementary data

Supplementary Figures and Tables. Image 1
