# Supplementary material for: Multiplex Ultrasensitive Genotyping of Patients with Non-Small Cell Lung Cancer for Epidermal Growth Factor Receptor (EGFR) Mutations by Means of Picodroplet Digital PCR
Source: eBioMedicine. 2017 Jun 7;21:86–93. doi: 10.1016/j.ebiom.2017.06.003 (PMC5514407; doi:10.1016/j.ebiom.2017.06.003)

Supplemental Text

Multiplex Ultrasensitive Genotyping of Patients with Non–Small Cell Lung Cancer for Epidermal Growth Factor Receptor (EGFR) Mutations by Means of Picodroplet Digital PCR

Supplementary Figure Legends

Supplemental Fig. 1. Serial dilutions of positive control plasmid were measured for each of the EGFR mutation. (A) Two-dimensional histogram of the 6-hexaplex assay is shown. **(B)** Individual experiments are displayed as diamond, square, or triangles, which correspond to three independent dilution series experiments, which were measured for each target. FAM, 6-carboxyfluorescein; TET, tetrachlorofluorescein.

Supplemental Fig. 2. False positive droplet events. For each of the 3 EGFR mutation targets, the distribution of false positives was measured from a pooled collection of wild type plasmid samples, commercial human normal genomic DNA, and genomic DNA from negative cell line. A Poisson model was applied to fit the false positive distribution. The table in the lower right reports the number of pooled experiments used in the limit

of blank (LOB) determined from the 95% confidence interval of the Poisson model fit (Bottom right panel).

Supplemental Fig. 3. False positive droplet events and amount of DNA. (A) Multiplex analysis of DNA isolated from a FFPE sample of EGFR L858R negative and exon19 deletion positive sample (left, input DNA was approximately 184.9 ng), EGFR L858R positive and exon19 deletion negative sample (middle, input DNA was approximately 114.7 ng) and EGFR exon19 deletion positive and T790M negative sample (right, input DNA was approximately 110.3 ng). (B) Correlation between the quantity of amplifiable DNA and the number of mutant alleles per assay for EGFR L858R (left), exon19 deletion (middle) and T790M (right).

Supplemental Fig. 4. False positive rate of multiplex ddPCR assay. For the EGFR L858R (A), exon19 deletion (B), or T790M (C) mutation targets, the distribution of false positives was measured from a pooled collection of mutation negative samples. A Poisson model was applied to fit the false positive distribution. The table in D reports the number of pooled experiments used in the limit of blank (LOB) determined from the 95% confidence interval of the Poisson model fit.

Supplemental Fig. 5. Analysis of an identical sample with duplex and multiplex assays. Multiplex analysis of DNA isolated from a FFPE sample of EGFR L858R mutant (A), exon19 deletion mutant (B) and KRAS mutant (C) samples with NSCLC. Duplex analyses of identical DNA samples from each patient are shown in right three panels.

Supplemental Fig. 6. Comparison of results obtained by multiplex and duplex analyses on fraction of wild type DNA. Compilation of the analysis of FFPE samples from patients with primary tumors mutated for EGFR L858R, exon19 deletion (ex19del) with/without T790M mutation.

Supplemental Table 1. Primers and probes used for droplet digital PCR (ddPCR).

Target mutation	Primers	Sequence
L858R	Forward	5'-GCAGCATGTCAAGATCACAGATT-3'
	Reverse	5'-CCTCCTTCTGCATGG TATTCTTTCT-3'
Exon19 del	Forward	5'-GCACCATCTCACAATTGCCAG-3'
	Reverse	5'-CACAGCAAAGCAGAAACTCACA-3'
T790M	Forward	5'-GCAGCATGTCAAGATCACAGA TT-3'
	Reverse	5'-CCTCCTTCTGCATGGTATTCTTTC T-3'

Target mutation	Probes	Reporter dye	Quenchers	Sequence	Concentration (nM)
L858R	Wild-type (2573T)	TET	ZEN/IABkFQ	5'-AGTTTGGCCAGCCCAA-3'	200
	L858R mutant (2573T>G)	TET	ZEN/IABkFQ	5'-AGTTTGGCCCGCCCAA-3'	40
	L858R mutant (2573T>G)	FAM	ZEN/IABkFQ	5'-AGTTTGGCCCGCCCAA-3'	200
Exon19 del	Wild-type	TET	ZEN/IABkFQ	5'-TATGTTGCTTCTCTTAATTCC-3'	200
	Reference	FAM	ZEN/IABkFQ	5'-CAGAAGGTGAGAAAGTT-3'	200
T790M	Wild-type (2369C)	TET	ZEN/IABkFQ	5'-T+CATC+A+C+GC/ZEN/A+GCTC-3'	200
	T790M mutant (2369C>T)	FAM	ZEN/IABkFQ	5'-T+CATC+A+T+GCA+GC+TC-3'	200

* + indicates Locked Nucleic Acids (LNA).

Supplemental Table 2. PCR conditions

Duplex: EGFR L858R		
**Cycle**	**Temperature (°C)**	**Hold**
1	95	10 min
	60	2 min
45	95	30 sec
	60	1 min
1	98	10 min
	10	∞

Duplex: EGFR exon19 deletion		
**Cycle**	**Temperature (°C)**	**Hold**
1	95	10 min
	60	2 min
45	98	30 sec
	60	2 min
1	98	10 min
	10	∞

Duplex: EGFR T790M		
**Cycle**	**Temperature (°C)**	**Hold**
1	95	10 min
45	95	15 sec
	58	15 sec
	60	45 sec
1	98	15 min
	10	∞

Multiplex		
**Cycle**	**Temperature (°C)**	**Hold**
1	95	10 min
45	95	30 sec
	58	15 sec
	60	75 sec
1	98	15 min
	10	∞

Supplemental Fig. 1

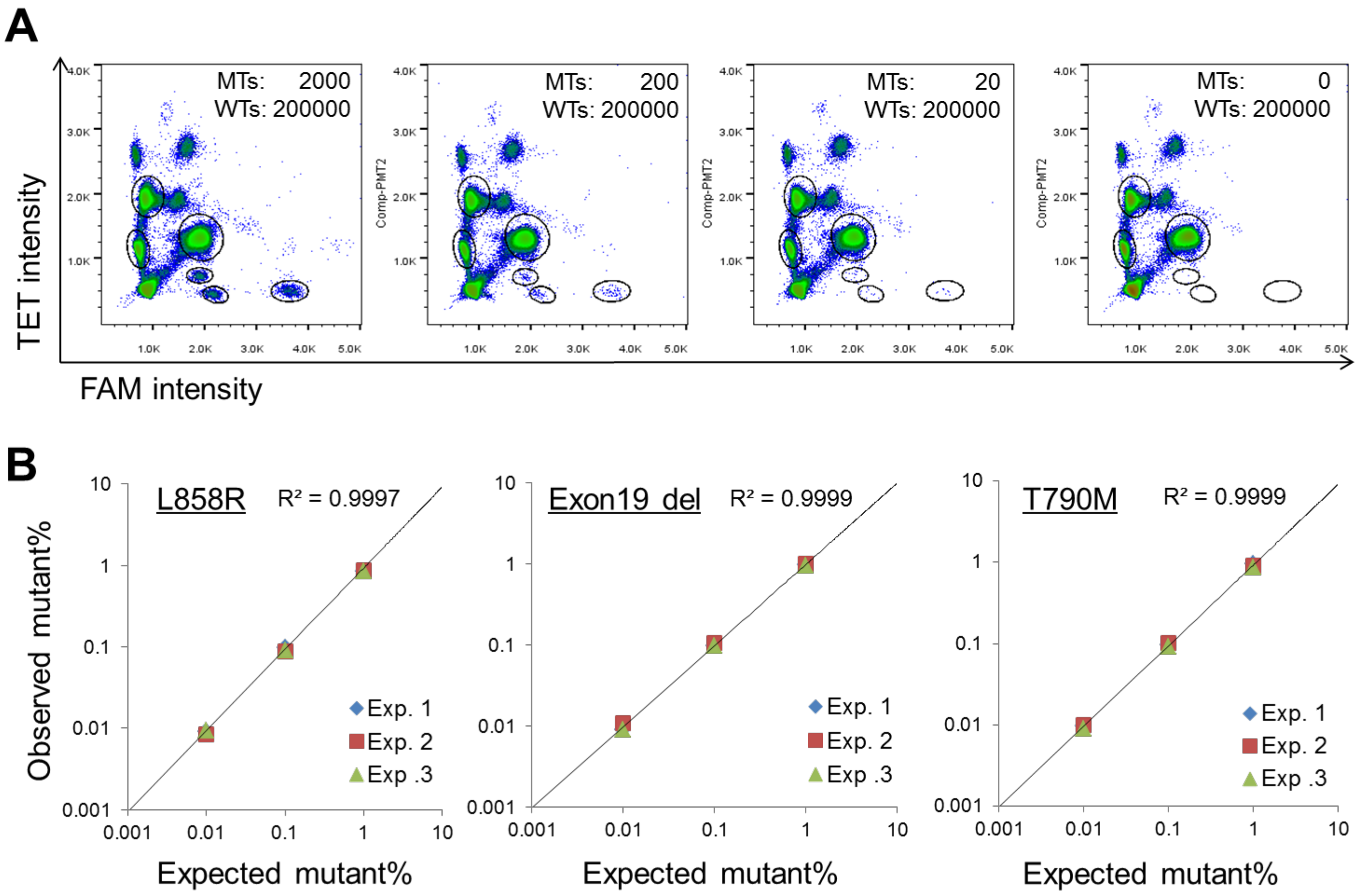

Supplemental Fig. 2

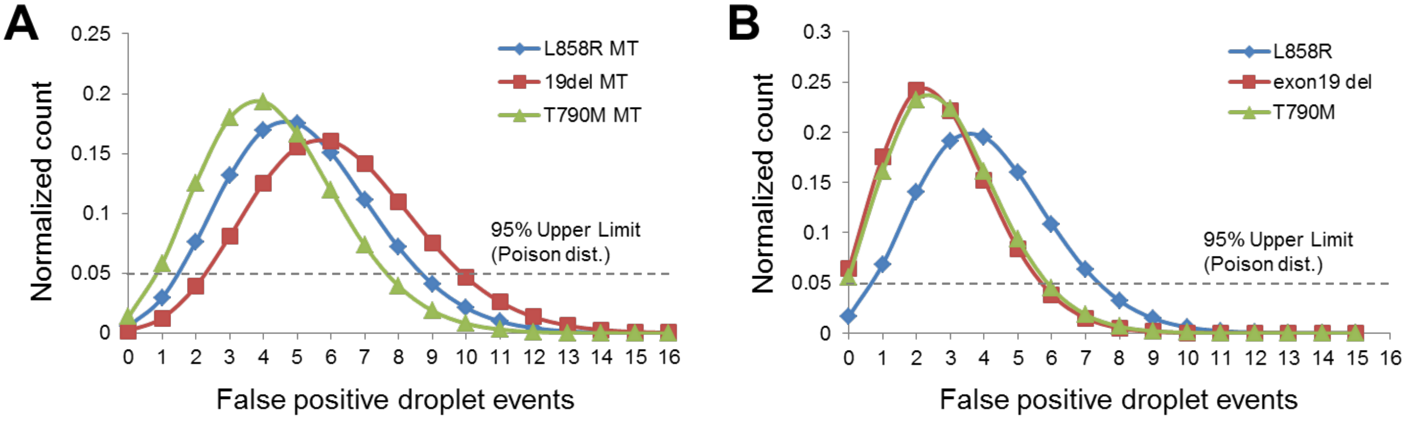

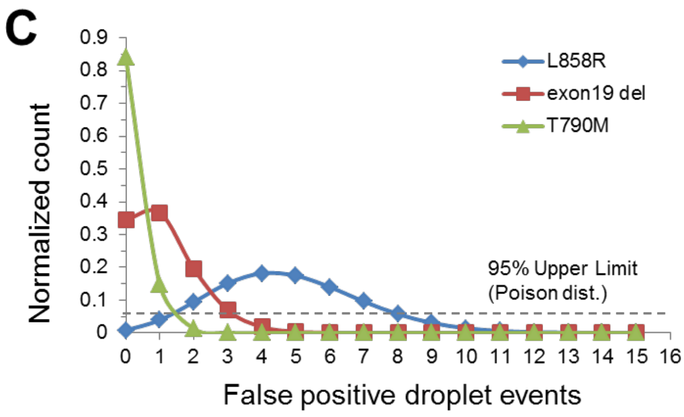

D

Template DNA (*n* = 8)	Limit of blank (LOB)/assay		
	L858R	Ex19 del	T790M
Wild-type plasmid	9	10	7
Human normal gDNA	8	6	6
A549 gDNA	9	4	2

Supplemental Fig. 3

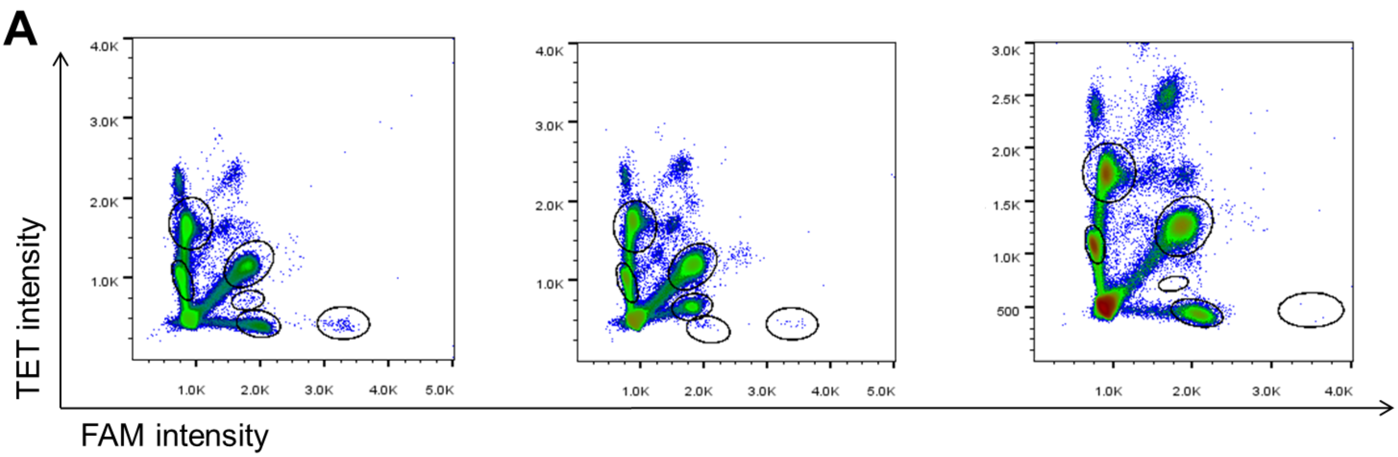

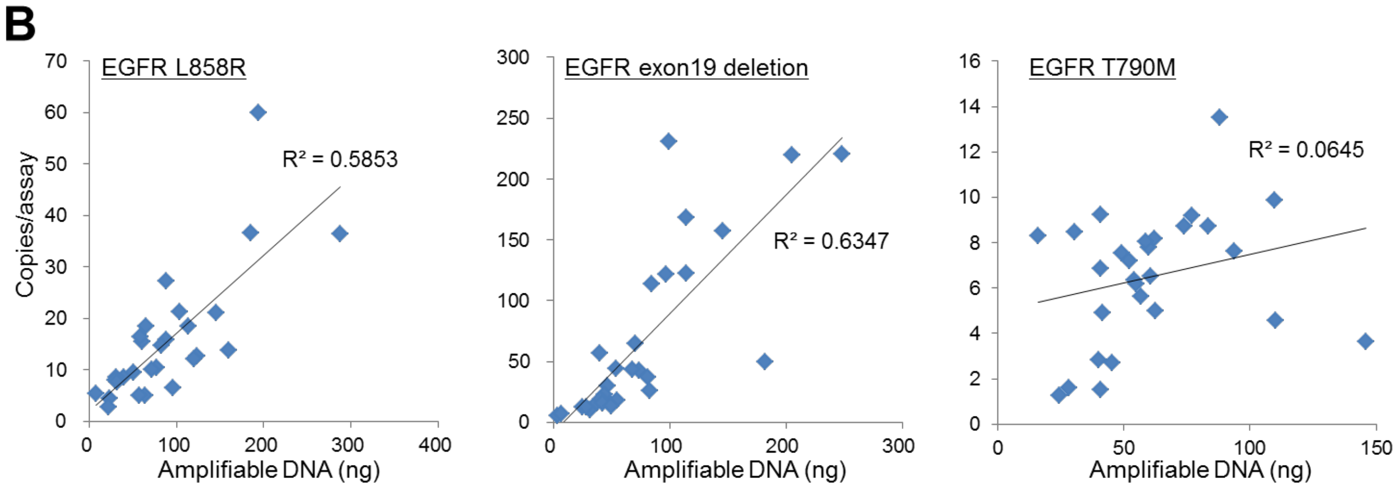

Supplemental Fig. 4

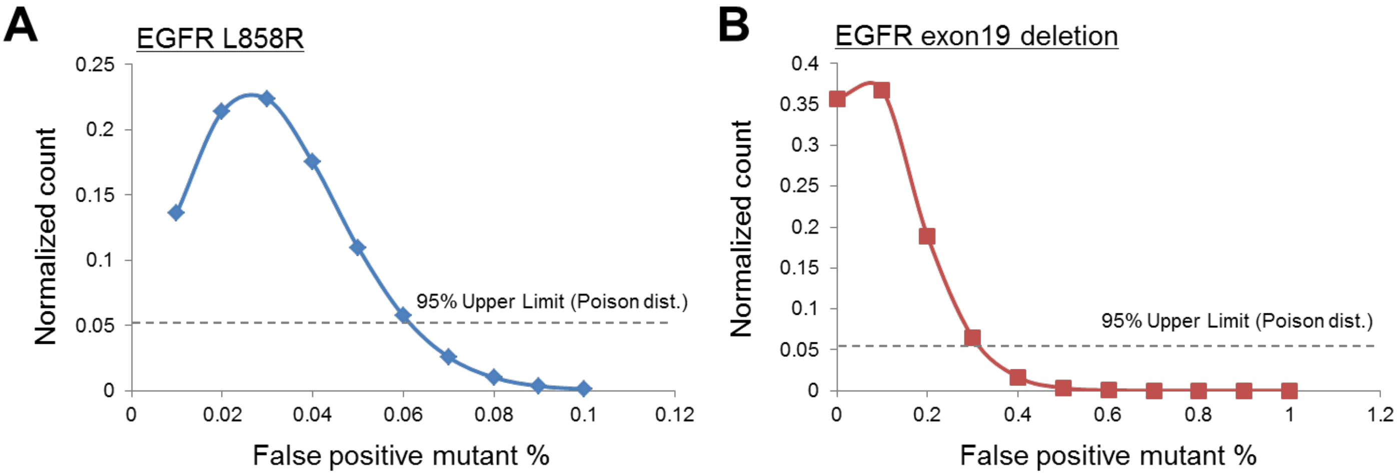

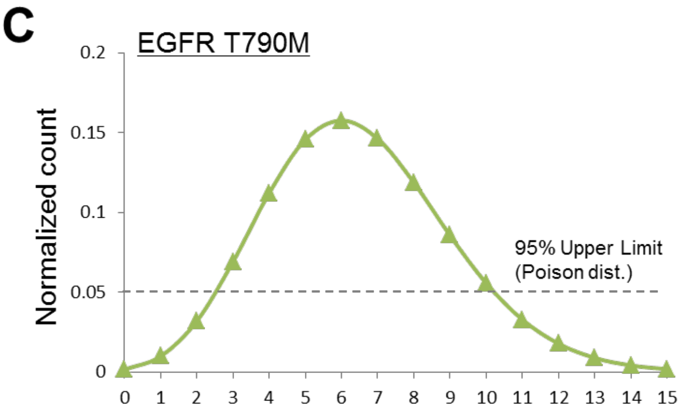

D

	Limit of blank (LOB)		
	L858R (*n* = 29)	Ex19del (*n*=28)	T790M (n=28)
Genome DNA from EGFR activation mutation negative samples	0.07%	0.4%	11 copies /assay

Supplemental Fig. 5

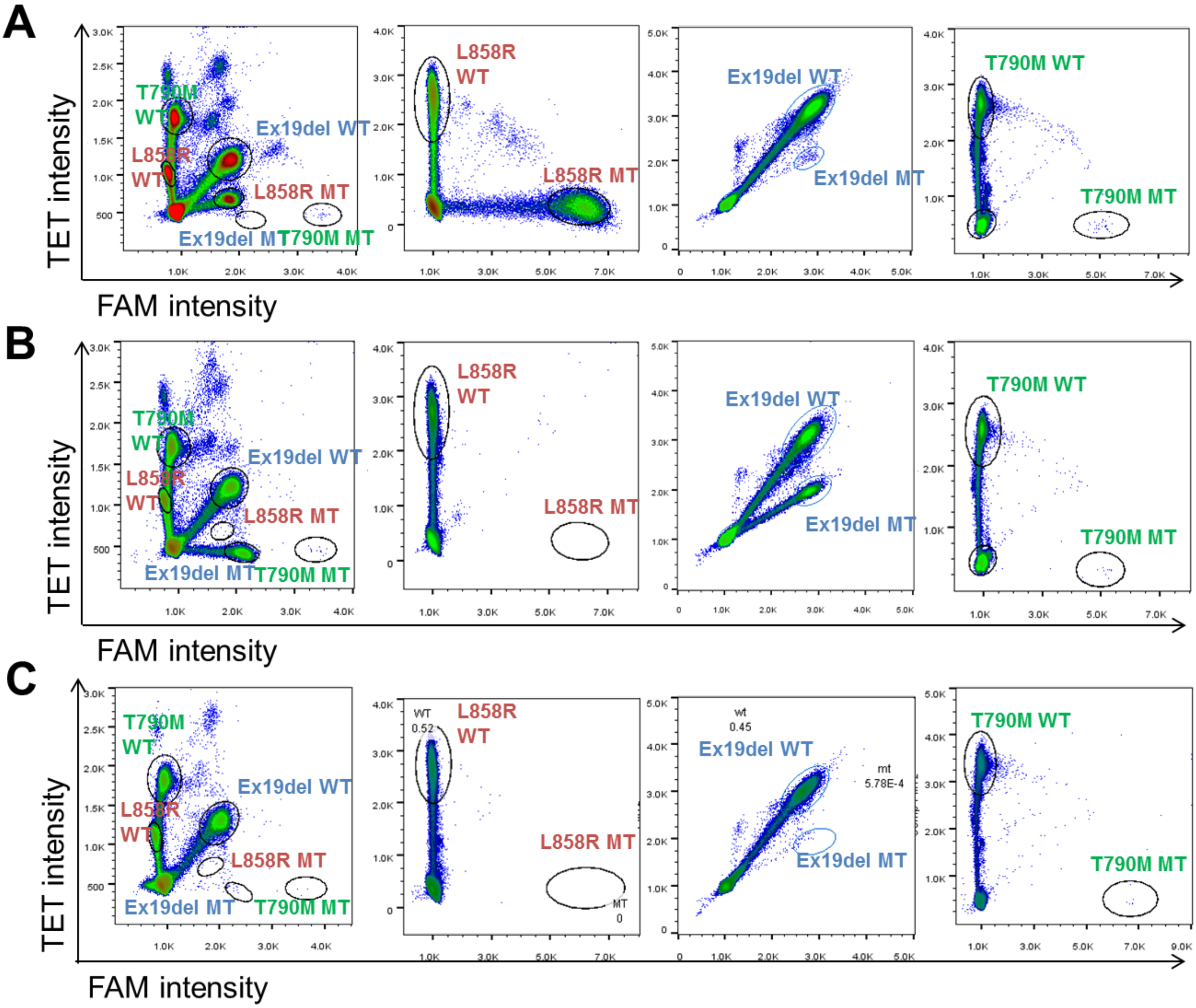

Supplemental Fig. 6

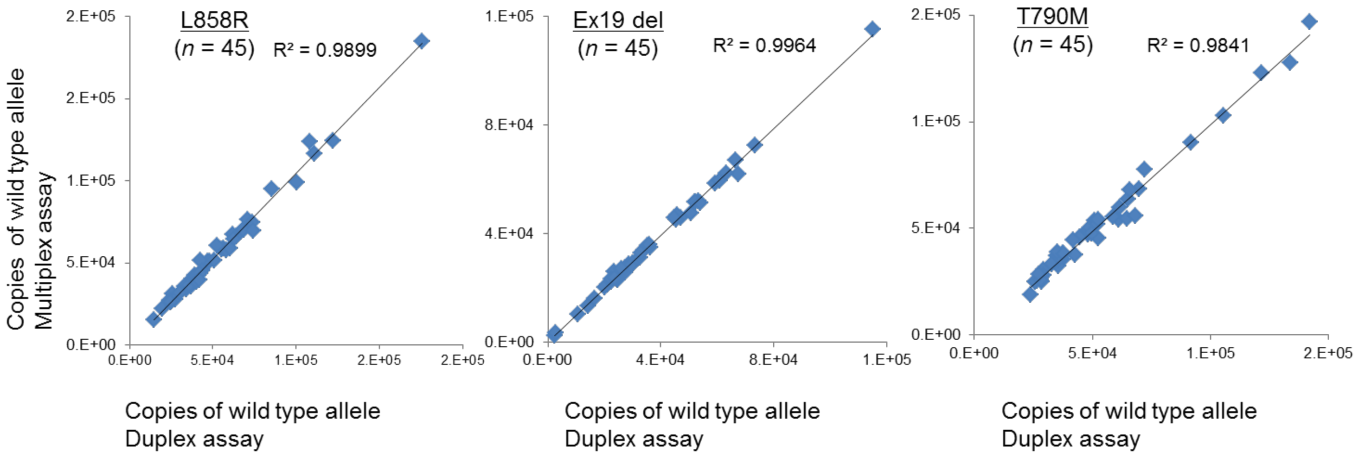

Supplement: Supplementary file 1 — Supplementary Figures and Tables. [file mmc1.pdf]
